# PROMIS Global-10 poorly correlates with legacy outcomes for patients undergoing hip arthroscopy

**DOI:** 10.1093/jhps/hnab033

**Published:** 2021-05-11

**Authors:** Jennifer Bido, Spencer W Sullivan, Matthew S Dooley, Danyal H Nawabi, Anil S Ranawat, Bryan T Kelly, Benedict U Nwachukwu

**Affiliations:** Department of Orthopaedic Surgery, Hospital for Special Surgery, New York, NY, USA

## Abstract

The Patient-Reported Outcomes Measurement Information System (PROMIS) Global-10 assesses generic-related quality of life, but has not been well studied in the orthopaedic literature. The purpose was to compare PROMIS Global-10 and legacy hip-specific patient-reported outcome measures (PROMs) in patients undergoing hip arthroscopy for femoroacetabular impingement syndrome (FAIS). This study included patients who underwent primary hip arthroscopy with complete preoperative and 6-month post-operative follow-up. PROMIS Global-10 Physical (PROMIS-P) and Mental (PROMIS-M) components, as well as the modified Harris hip score (mHHS) and International Hip Outcome Tool-33 (iHOT-33) were assessed. PROM analysis included: post-operative changes, correlations, floor and ceiling effects and responsiveness. Final analysis included 112 patients. Average age and body mass index were 36.1±11.7 years and 24.8±3.9 kg/m^2^, respectively. All 6-month PROMs, except PROMIS-M, were significantly improved compared to preoperative level (*P*<0.02). Preoperatively, PROMIS-P was poorly correlated with mHHS and iHOT-33 (*r*_s _<0.4) whereas PROMIS-M was only poorly correlated with iHOT-33 (*r*_s _<0.4, 95% CI of 0.02–0.37). Post-operatively, the iHOT-33 was poorly correlated with both PROMIS measures (*r*_s _<0.4). The mHHS was fairly correlated with both PROMIS measures (*r*_s _<0.6) post-operatively. The effect sizes for mHHS and iHOT-33 were high (*d*=1.2 and 1.40, respectively), whereas the effect sizes for PROMIS Global-10 were small (*d*<0.3). PROMIS Global-10 demonstrated lower effect sizes and poor to fair correlation with legacy hip-specific PROMs, and appears to have a limited role in the assessment of patients undergoing hip arthroscopy for FAIS. Therefore, the PROMIS Global-10 may have a limited role in assessing patients with FAIS.

## INTRODUCTION

Patient-reported outcome measures (PROMs) are widely used within the field of orthopaedics to quantify the effectiveness of a surgical intervention’s ability to improve patients’ quality of life [1–4]. Historically, PROMs were often joint specific, which resulted in multiple measures per joint and disease. There are many prior studies in the literature that establish the validity of various joint specific and general quality of life PROMs for the assessment of hip arthroscopy outcomes [5–7]. Subsequently, studies then focused on comparing the psychometrics of different measures in order to determine which were the most appropriate [4, 8]. Despite previous contributions on this topic, optimal outcome measures have not been standardized for each joint and general quality of life.

One of the most widely used legacy PROMs for hip conditions is the modified Harris hip score (mHHS) [4]. The mHHS assesses elements of pain and function similar to the original Harris hip score [9, 10]. Additionally, the International Hip Outcome Tool-33 (iHOT-33), which measures hip related function, sport, job and emotional limitations in 33 questions, is widely used [6, 11]. Both the mHHS and iHOT-33 have had their reliability, validity, responsiveness, interpretability and floor and ceiling effects appraised by prior studies [12, 13]. Prior analyses determined that while the mHHS is more widely used, the iHOT-33 had superior psychometric properties [4, 8]. In addition to these hip-specific PROMs, general health assessments are also used in the evaluation of hip pathology. The Short-form 36 (SF-36) health survey is the most commonly used general health assessment in the hip preservation literature [14]. Specifically, The SF-36 is a measure of health-related quality-of-life, which utilizes 36 items that cover eight health domains [14]. Its ability delineate post-operative changes has been documented and the physical component score has been found to have good correlation with the mHHS in patients undergoing hip arthroscopy [7, 15]. Notably, these PROMs and many others are routinely combined together to evaluate patients with femoroacetabular impingement syndrome (FAIS). However, the multitude of PROMs can be cumbersome to patients and can create inconsistencies when comparing studies.

In an effort to decrease the patient’s burden of answering multiple PROMs, the Patient-Reported Outcomes Measurement Information System (PROMIS) was developed by the National Institutes of Health [16]. This system aims to provide a single, generalizable and validated PROM that can be used for various diseases and conditions. A shortened version of the PROMIS, entitled the PROMIS Global-10 has gained recent traction within the orthopaedic literature [17–19]. This version assesses physical function, fatigue, pain, emotional distress and social health with only q10 questions, producing mental and physical health scores [20]. While PROMIS computer adaptive testing, such as PROMIS physical function, is meant to be a companion to the disease-specific legacy PROMs, the PROMIS Global-10 was created as a non-computer adaptive, general health assessment tool. That makes it similar to the SF-36 health survey and its shorter version, the SF-12, which are meant to be used across different orthopaedic conditions, including FAIS [21–23]. Although ideally this shortened PROM could be an efficient way to decrease question burden, a thorough analysis of its utility is needed prior to retiring the use of legacy PROMs.

Within the hip preservation literature, recent studies comparing the performance of the legacy PROMs relative to PROMIS computer adaptive testing have shown psychometric deficiencies in the latter [24, 25]. Specifically, good to excellent correlations have been reported between preoperative legacy PROMs and PROMIS physical function for patients with FAIS [25]. However, the legacy PROMS were noted to be more responsive. Notably, there is a paucity of data within orthopaedic literature extending this psychometric analysis to the PROMIS Global-10. As such, the aims of this study are (i) to compare pre and post-operative PROMIS Global-10 and mHHS and iHOT-33 scores in patients undergoing hip arthroscopy for FAIS, (ii) assess the correlation between the PROMIS Global-10 and mHHS and iHOT-33 and (iii) analyse the psychometric properties of the PROMIS Global-10. We hypothesized that there would be poor correlations between PROMIS Global-10 and legacy PROMS both pre and post-operatively, and that the legacy PROMs would demonstrate higher responsiveness.

## MATERIALS AND METHODS

### Study design and patient selection

This study and respective institutional registry of four fellowship-trained surgeons was approved by the hospital Institutional Review Board. A retrospective analysis from an institutional registry was conducted on patients that underwent primary hip arthroscopy for treatment of FAIS between December 2016 and August 2019. Inclusion criteria for this study included: clinical and radiographic diagnosis of symptomatic FAIS, operative treatment with primary hip arthroscopy and completion of preoperative and 6-month follow-up surveys. Exclusion criteria included: hip arthroscopy for an indication other than FAIS, revision hip arthroscopy and any procedures not identified under the following current procedural terminology (CPT): CPT-29862 (with chondroplasty, abrasion arthroplasty and/or resection of labrum), CPT-29863 (with synovectomy), CPT-29914 (with femoropasty), CPT-29915 (with acetabuloplasty) and CPT-29916 (with labral repair). There were 623 patients in the registry with a diagnosis of FAIS who underwent a primary hip arthroscopy and completed all preoperative questionnaires. Of those patients, 112 (18%) patients completed all post-operative questionnaires.

### Assessment of outcomes

Demographic information including age, sex, body mass index (BMI) and surgical procedure were extracted from the registry for each patient. The legacy hip-specific PROMs included the mHHS [9], and the iHOT-33 question version [6]. Additional primary outcome measures included the PROMIS Global-10 Health Score, both Physical (PROMIS-P) and Mental (PROMIS-M) components [20]. The components of the PROMIS Global-10 are initially reported as a raw value, which is then used to derive a T-score. The T-score is standardized such that a 50 represents the average for the US general population with a standard deviation (SD) of 10.

### Statistical analysis

Descriptive statistics, such as means with SDs and frequency statistics were used to report baseline characteristics. Changes in 6-month follow-up scores compared to preoperative scores were detected using paired *t*-tests. Additionally, comparisons between preoperative and post-operative cohorts were made utilizing paired *t*-tests and chi-square tests. Statistical significance was set at an α ≤ 0.05. All analyses were performed using SAS Software version 9.4 (SAS Institute).

The floor and ceiling effect for the mHHS, iHOT-33, PROMIS-P and PROMIS-M were also analysed. The mHHS and iHOT-33 are scored from 0 to 100; therefore, the presence of a ceiling or floor effect was defined as any percentage ≥15% of the study population in the top or bottom 5% [26, 27]. For both PROMIS measures, the presence of a ceiling or floor effects was defined as any percentage ≥15% of the study population in the top or bottom 5^th^ percentile [26, 27].

In order to determine the correlations between legacy PROMs and the PROMIS scores, all data were analysed and classified as either parametric or nonparametric using the Shapiro–Wilk test of normality. Given that all the measures were non-parametric (*P* < 0.05), Spearman coefficient analysis was then conducted to assess the correlations [28]. For this study, we used the following coefficient classifications: excellent (>0.80), very good (0.71–0.80), good (0.61– 0.70), fair (0.41–0.60) and poor (0.21–0.40) [29].

To directly compare the responsiveness between PROMs, the effect size and relative efficiency (RE) were calculated for each PROM [30–32]. Effect size, or Cohen’s *d* is defined as the absolute difference in the mean change score for each PROM divided the by the pooled SD for that PROM tool [32–34]. For example, *d* will increase by 1 as the magnitude of the preoperative to post-operative change increases by a SD. For this study, we used the following effect size classification: large (>0.80), moderate (0.50–0.79) and small (0.20–0.49) [32–34]. RE is used to directly compare responsiveness between PROMs. In order to calculate RE, first paired *t*-tests comparing the preoperative and post-operative scores for each specific PROM are conducted. RE is then determined by dividing the *t*-score from one PROM by the *t*-score of another PROM and then squaring the result [4, 30, 31]. The first PROM would be considered ‘more responsive’ than the second PROM tool if the RE ≥1 [4, 30, 31].

## RESULTS

### Demographic characteristics

Final analysis included 112 patients (59.8% female). Average age and BMI were 36.1 ± 11.7 years and 24.8 ± 3.9 kg/m^2^, respectively. In addition to hip arthroscopy most patients had a labral repair (92.9%), a femoroplasty (72.3%) and/or chondroplasty, abrasion arthroplasty and/or resection of labrum (52.7%). Baseline PROM scores are stated in Table I. The PROMIS physical T-score was not significantly different than that of the general US population (48.1 versus 50; SD 6.9, *P* = 0.10). However, the PROMIS mental T-score was higher than that of the general US population (55.7 versus 50; SD 7.9, *P* = 0.0001). This indicates that while this cohort’s physical function was similar to that of the general US population, their mental health scores were higher.

**Table I.** Analysis of pre- and post-operative reported outcomes

Instrument	Scale	Baseline mean (SD)	Follow-up mean (SD)	*P*-value
PROMIS Global-10				
Physical raw score	4–20	15.0 (2.3)	15.7 (2.4)	0.009
Mental raw score	4–20	16.4 (2.7)	15.8 (3.0)	0.06
Physical T-score	0–100	48.1 (6.9)	50.1 (7.6)	0.008
Mental T-score	0–100	55.7 (7.9)	53.6 (8.6)	0.012
mHHS	0–100	62.3 (12.5)	79.9 (16.0)	<0.0001
iHOT-33	0–100	41.2 (17.1)	69.0 (22.3)	<0.0001

PROMIS, Patient-Reported Outcomes Measurement Information System; mHHS, modified Harris hip score; iHOT-33, International Hip Outcome Tool-33.

### Post-operative changes

All outcome measures, besides PROMIS mental raw score, were significantly different at 6 months compared to the preoperative level (*P* < 0.02) (Table I). Both mHHS (62.3 ± 12.5 versus 79.9 ± 16, *P* < 0.0001) and iHOT-33 (41.2 ± 17.1 versus 69.0 ± 22.3, *P* < 0.0001) scores were significantly increased at 6 months post-operatively, indicating improvement. PROMIS physical T-score also had a significant improvement (48.1 ± 6.9 versus 50.1 ± 7.6, *P* = 0.008). Alternatively, PROMIS mental T-score significantly decreased post-operatively (55.7 ± 7.9 versus 53.6 ± 8.6, *P* = 0.012), which suggests a negative change in mental health and quality of life. Analysis of the Global-10 raw scores yielded smaller mean pre to post-operative differences, resulting in a significant change in the physical score but no difference in the mental score.

### Correlation analysis

Preoperatively, both the PROMIS-P and PROMIS-M components (T-score and raw scores) were either poorly correlated with all legacy PROMs (*r*_s__ _< 0.4) or not correlated at all (Table II). Preoperatively, PROMIS physical T-score and raw score were poorly correlated with mHHS and iHOT-33 (*r*_s__ _< 0.4). PROMIS mental T-score and raw score were not correlated with mHHS *(r*_s__ _< 0.2). PROMIS mental T-score had poor correlation with iHOT-33 (*r*_s__ _= 0.2, 95% CI of 0.02–0.37), while PROMIS mental raw score had no correlation with iHOT-33 (*r*_s__ _= 0.16, 95% CI −0.03–0.33).

**Table II.** Correlation analysis of pre-operative reported outcomes

	PROMIS physical raw score *r*_s_ (95% CI)	PROMIS mental raw score *r*_s_ (95% CI)	PROMIS physical T-score *r*_s_ (95% CI)	PROMIS mental T-score *r*_s_ (95% CI)
mHHS	0.37 (0.20–0.52)	0.11 (−0.07–0.29)	0.35 (0.18–0.50)	0.10 (−0.09–0.28)
iHOT-33	0.40 (0.23–0.55)	0.16 (−0.03–0.33)	0.38 (0.21–0.53)	0.20 (0.02–0.37)

Post-operatively, PROMIS physical T-score demonstrated fair correlation with mHHS (*r*_s__ _= 0.59, 95% CI of 0.45–0.70) and poor correlation with iHOT-33 (*r*_s__ _= 0.21, 95% CI of 0.02–0.38) (Table III). PROMIS mental T-score showed fair correlation with the mHHS (*r*_s__ _= 0.45, 95% CI of 0.29–0.59) and poor correlation with iHOT-33 (*r*_s__ _= 0.25 95% CI of 0.07–0.42). Analysis of the Global-10 raw scores yielded similar results.

**Table III.** Correlation analysis of post-operative reported outcomes

	PROMIS physical raw score *r*_s_ (95% CI)	PROMIS mental raw score *r*_s_ (95% CI)	PROMIS physical T-score *r*_s_ (95% CI)	PROMIS mental T-score *r*_s_ (95% CI)
mHHS	0.61 (0.47–0.71)	0.46 (0.30–0.59)	0.59 (0.45–0.70)	0.45 (0.29–0.59)
iHOT-33	0.22 (0.03–0.38)	0.25 (0.07–0.42)	0.21 (0.02–0.38)	0.25 (0.07–0.42)

Values indicate *r*_s_ value and 95% confidence interval (95% CI). The correlations with the PROMIS raw score and PROMIS T-score are similar.

### Analysis of responsiveness

No floor effect was observed for any measure and the mHHS was the only outcome measure with a ceiling effect (Table IV). Namely, 25% of mHHS scores were in the top 5% of possible scores. The effect size analysis demonstrated that the PROMIS measures had mean score differences between preoperative status and 6-month follow-up that were nearly one-tenth the size of legacy PROM mean differences (Table V). However, they had smaller variation as shown by the smaller SDs. The effect sizes for mHHS and iHOT-33 were high (*d* = 1.2 and 1.4, respectively), whereas the effect sizes for PROMIS-P T-score and PROMIS-M T-score were small (*d* = 0.28 and 0.26, respectively). Analysis of the PROMIS raw scores yielded similar results.

**Table IV.** Floor and ceiling effects

	Floor (%)	Ceiling (%)
mHHS	0.00	25.00
iHOT-33	0.00	11.60
PROMIS physical raw score	0.00	1.80
PROMIS mental raw score	0.00	13.40
PROMIS physical T-score	0.00	0.00
PROMIS mental T-score	0.00	0.00

Floor effects were calculated preoperatively. Ceiling effects were calculated post-operatively.

**Table V.** Pre- versus post-operative outcome score difference and pooled effect size

	Mean score difference	Pooled SD	Pooled effect size (*d*)
mHHS	17.5	14.3	1.2
iHOT-33	27.9	19.8	1.4
PROMIS physical raw score	0.63	2.3	0.27
PROMIS mental raw score	0.55	2.9	0.19
PROMIS physical T-score	2.	7.3	0.28
PROMIS mental T-score	2.1	8.3	0.26

The analysis of responsiveness demonstrated stark differences between the legacy PROMs and the PROMIS measures (Table VI). The mHHS is slightly more responsive than iHOT-33 (RE = 1.02) and much more responsive than both PROMIS T-scores (RE = 18.4 for PROMIS-P and 20.2 for PROMIS-M). The iHOT-33 is also more responsive than both PROMIS T-scores (RE = 18.0 for PROMIS-P and 19.7 for PROMIS-M). The PROMIS physical T-score was more responsive than the PROMIS mental T-score (RE = 1.1). The mHHS was the most responsive measure while the PROMIS mental T-score was the least responsive overall.

**Table VI.** RE between pre- and 6-months post-operative outcome measures

	mHHS	iHOT	PROMIS physical T-score	PROMIS mental T-score
mHHS	—	1.02	18.4	20.2
iHOT	0.98	—	18.0	19.7
PROMIS physical T-score	0.05	0.06	—	1.1
PROMIS mental T-score	0.05	0.05	0.9	—

A value of >1 signifies that the measure on the left column is more responsive than then the PROM in the top row.

### Analysis of missing data

Of the 623 patients who met all inclusion criteria, 112 patients completed all 6-month follow-up questionnaires. A comparison of preoperative characteristics and PROM scores between the final cohort and those who did not complete follow-up surveys was tabulated (Table VII). Although patients who lacked follow-up were significantly younger (32.9 ± 11.2 versus 36.1 ± 11.2, *P* = 0.007), there were no other significantly different demographic characteristics between patients lacking follow-up and the study cohort with complete follow-up. Notably, all baseline PROMs were not statistically different between these groups.

**Table VII.** Analysis of missing data at baseline measures

	Incomplete follow-up (*N*=511)	Complete follow-up (*N*=112)	
	Mean (SD)	Mean (SD)	*P*-value
Age (years)	32.9 (11.2)	36.1 (11.7)	0.007
Females (*N*, %)	256 (50.10%)	67 (59.8%)	0.06
BMI	24.5 (4.4)	24.8 (3.9)	0.51
PROMIS Global-10			
Physical raw score	15.2 (2.2)	15.0 (2.3)	0.55
Mental raw score	16.5 (2.8)	16.4 (2.7)	0.66
Physical T-score	49.1 (6.9)	48.1 (6.9)	0.19
Mental T-score	55.8 (8.4)	55.7 (7.9)	0.91
mHHS	61.8 (13.0)	62.3 (12.5)	0.69
iHOT-33	39.5 (17.2)	41.2 (17.1)	0.34

## DISCUSSION

This study analysed the utility of the PROMIS Global-10 as a post-operative PROM relative to legacy hip-specific PROMs at six-month follow-up in a cohort of patients treated with hip arthroscopy for FAIS. Our analysis demonstrated a significant change at follow-up in every measure except PROMIS mental raw score. The PROMIS Global-10 measures demonstrated poor to fair correlation with the legacy PROMs both preoperatively and post-operatively, often showing no statistical correlation. Additionally, we found no floor effect for any measure and the mHHS was the only outcome measure with a ceiling effect. In terms of the responsiveness analysis, the effect sizes for mHHS and iHOT-33 were up to 10 times higher than the effect sizes for PROMIS physical and PROMIS mental scores. Finally, the mHHS and iHOT-33 had similar responsiveness and were both markedly more responsive than the PROMIS Global-10 components.

Although there are no studies assessing PROMIS Global-10 scores in FAIS patients, prior studies have investigated the use of PROMIS computer adaptive tests (CAT) in hip preservation patients. Kollmorgen *et al.* [24] looked at the PROMIS CAT and legacy PROM scores in a cohort of patients with different hip conditions and demonstrated strong correlations between the PROMIS physical function score both pre- and post-operatively. Nwachukwu *et al.* [25] conducted a similar investigation but focused on preoperative scores in FAIS patients. They demonstrated good to excellent correlation between the PROMIS physical function score and the hip-specific legacy PROMs. Neither study found any floor or ceiling effects [24, 25]. As such, these studies concluded that the PROMIS physical function score may be utilized for the analysis of hip preservation interventions [24, 25]. Given the success of PROMIS CAT in this population, it is notable that the PROMIS Global-10 does not produce similar results in this cohort. This may be due in part to the difference in the utility of general health PROMs like the Global-10 and disease-specific measures, such as the PROMIS CAT, in patients with FAIS. However, general health measures, such as the SF-36, are commonly used to evaluate patients after hip arthroscopy as they are able to delineate post-operative changes [7]. Prior research has shown good correlation between the SF-36 and mHHS *(r* > 0.7), concluding that both should be used when evaluating patients after hip arthroscopy [7]. Therefore, if the Global-10 is meant to be the PROMIS equivalent of the SF-36 then it should demonstrate a correlation with previously established hip-specific PROMs.

Despite a paucity of research on the use of PROMIS Global-10 in patients with FAIS, a more extensive literature search does provide insight into its utility. However, all of the current studies have focused on its applicability for upper extremity conditions. Nicholson *et al.* [18] looked at the Global-10 compared to legacy PROMs for rotator cuff disease. This preoperative comparison demonstrated good to excellent correlation between PROMIS-P and legacy PROMs and poor correlations between PROMIS-M and legacy PROMs [18]. A similar study conducted by Kahan *et al.* [17] for lateral epicondylitis demonstrated good to excellent correlation between PROMIS-P and legacy PROMs and poor to good correlations between the PROMIS-M and legacy PROMs preoperatively. Finally, Saad *et al.* [19] conducted a preoperative comparison between the Global-10 and legacy PROMs for shoulder arthritis. They demonstrated poor to good correlations with PROMIS-P and legacy PROMs, compared to poor correlations with PROMIS-M [19]. Numerous points distinguish these previous publications from our study. Given that our study focuses on FAIS patients, the legacy PROMs utilized for comparison are not the same. Additionally, these studies were limited to preoperative comparisons and did not expand their psychometric analysis to responsiveness of each measure. Notably, these upper extremity studies demonstrated good to excellent correlations between PROMIS-P and their specific legacy PROMs, whereas our study only demonstrated poor to fair correlations. The fact that these studies concluded that the PROMIS Global-10 was a reliable PROM, while our study delineates its faults, emphasizes the need for disease-specific evaluation of measures prior to mass adaptation.

Our study fills a gap within the literature by illustrating the psychometrics of post-operative PROMIS Global-10 scores compared to hip-specific legacy PROMs for FAIS patients. The poor correlations between the PROMIS Global-10 scores and the hip-specific legacy PROMs suggest that the Global-10 may have a lower utility in the assessment of these patients. Although these shorter measures likely decrease the question burden that patients face, they are limited in their ability to delineate the patient’s experience. While the lack of improvement in PROMIS-M is likely due to the high baseline mental health status of our cohort, the change in PROMIS-P was also minimal and unlikely to be clinically significant. This is seen in the smaller effect sizes and RE of the PROMIS components relative to the legacy PROMs. Given the lack of data on post-operative PROMIS Global-10 scores, we cannot compare the responsiveness findings from this study to the literature. However, the responsiveness of the legacy PROMs is well established. Prior studies specific to hip preservation have demonstrated that the iHOT-33 and mHHS have satisfactory to excellent responsiveness [4, 8]. Additionally, they demonstrated that whereas the iHOT-33 has no ceiling or floor effects in this cohort, the mHHS has a 24% ceiling effect [4, 8]. Our study further confirms these findings, by demonstrating higher responsiveness for the iHOT-33 and mHHS, as well as only showing a 25% ceiling effect in the mHHS.

Limitations of this study included lack of PROMIS CAT and SF-36 data as this would have allowed direct comparisons with the Global-10. Additionally, this study is limited to 6-month post-operative follow-up, which may be too early to detect changes in the Global-10. However, given that prior studies have indicated that >50% of FAIS patients reached a minimally important clinical difference 6 months after surgery, we propose that the Global-10 should be able to delineate a difference [35]. Additionally, other hip procedures have shown statistically significant improvements in the SF-12 mental and physical component scores at 6-month follow-up, which could support improvements in other general health measures, including PROMIS Global [36]. Another limitation is that the high preoperative physical and mental Global-10 scores may have limited the ability to detect post-operative improvement. Finally, the low rate of post-operative survey completion is a significant limitation. This may due to a combination of factors, such as high patient question burden and interruptions in administrative reminders to patients about the surveys. However, the final cohort in this study had similar baseline characteristics to the larger preoperative cohort. Therefore, we propose that the results from this cohort are representative of the larger preoperative cohort.

## CONCLUSION

PROMIS Global-10 has poor correlation with legacy hip-specific PROMs in patients undergoing surgery for FAIS. Legacy hip PROMs had higher effect sizes and were more responsive than the PROMIS Global-10 in post-operative patients with FAIS. PROMIS Global-10 appears to have a limited role in disease-specific outcome assessment for patients with FAIS.
